# Editorial: Chang and Eng.

**Published:** 1874-03

**Authors:** 


EDITORIAL.—*Chang and Eng.*—In the present article it is not purposed to give an elaborate history of these famous twins, but only to put on record certain well-ascertained facts of physiological interest in regard to their life, to give all that can be ascertained as to the circumstances of their death, and to offer a brief history of the manner in which their bodies were brought to Philadelphia.

Chang and Eng Bunker were born in Siam, in the year 1811, but lived for many years in North Carolina, where they were married, and raised families of children, Chang being the father of ten, Eng of nine. Dr. Joseph Hollingsworth, to whom we are indebted for the information given in this article as to their habits of life and the circumstances of their death, states that he has known them as residents in the neighborhood of Mount Airy, North Carolina, for some twenty years, during which time he has acted as their family physician. Chang, who is said to have derived his name from the Siamese word for "left," was the left of the pair, and was much smaller and more feeble than his brother Eng, whose name signifies "right." Their habits were very active. During the latter part of their life they and their families lived in two houses, about a mile and a half apart, and it was an inflexible rule that they should pass three days alternately at each house. So determinedly was this alternation maintained that sickness and death in one family had no effect upon the movements of the father, and a dying or dead child was on one occasion left in obedience to it; indeed, Dr. Hollingsworth is very positively of the opinion that the death of the twins themselves was the result of this rule, or, at least, was materially hastened thereby. This will be made apparent hereafter.

The evidences during life that the twins were physiologically distinct entities were very numerous and apparent. They were different in form, tastes and disposition; all their physical functions were performed separately and unconnectedly. What Chang liked to eat, Eng detested. Eng was very good natured, Chang cross and irritable. The sickness of one had no effect upon the other, so that while one would be suffering from fever, the pulse of the other would beat at its natural rate. The twins not rarely suffered from bilious attacks, but one never suffered at the same time as the other; a circumstance which seems somewhat singular in view of the close connection which the post-mortem has shown to exist between the livers of the pair. Chang drank pretty heavily—at times getting drunk, but Eng never felt any influence from the debauch of his brother—a seemingly conclusive proof that there was no free interchange in their circulations.

The twins often quarrelled, and of course, under the circumstances, their quarrels were bitter. They sometimes came to blows, and on one occasion came under the jurisdiction of the courts. After one of these difficulties, Chang and Eng applied to Dr. Hollingsworth to separate them, stating that they could not live longer together. Eng affirmed that Chang was so bad that he could live no longer with him; and Chang stated that he was satisfied to be separated, only asking that he be given an equal chance with his brother, and that the band be cut exactly in the middle. But as Dr. Hollingsworth advised very decidedly against this, and declined to interfere, cooler counsels prevailed.

In August, 1870, Chang suffered from a paralytic stroke, from which he never fully recovered, and during the last year of his life he several times said to Dr. Hollingsworth, "We can't live long."

On the Thursday evening preceding their death, the time having arrived for their departure from the house at which they were staying, the twins rode a mile and a half in an open wagon. The weather was very cold, the night being the severest of the winter. Chang had been complaining for some days of cough, with distress and actual pain in the chest. He was so unwell that his wife thought he would be unable to bear the trip; but he finally went. On Friday morning Chang reported that he felt better, but that in the night he had had such severe pain in the chest, and so much distress, that he thought he should have died.

The twins slept in a room by themselves, or with only a very young child present; and some time in the course of Friday night they got up and sat by the fire. As they were accustomed to do this frequently, nothing was thought of it by those of the family who saw them, even though they heard Eng saying he was sleepy and wanted to retire, and Chang insisting on remaining up, stating that his breathing was so bad that it would kill him to lie down. Finally, however, the couple went to bed again, and after an hour or so the family heard some one call. No one went to the twins for some little time, and, when they did go, Chang was dead, and Eng was awake. He told his wife that he was very "bad off," and could not live. He complained of agonizing pain and distress, especially in his limbs. His surface was covered with a cold sweat. At his request his wife and children rubbed his legs and arms, and pulled and stretched them forcibly. This was steadily continued until he went into a stupor, which took place about an hour after the family were alarmed. The stupor continued up to death: according to the statements of the family, there were no convulsions.

Dr. Hollingsworth did not reach the house until after the death of both of the twins. He found the wives, and especially the children, averse to any post-mortem being made, but, after much persuasion, obtained permission to put the bodies in a position to be preserved until he could obtain some one from Philadelphia to perform the autopsy. He placed the bodies, after they had been thoroughly cooled, in a coffin, which was put in a wooden box, which was, in its turn, enclosed in tin; the whole being buried in a dry cellar in such a way as to be imbedded in charcoal.

As bearing upon the question, What was the cause of the death of Eng? it is important to state that Dr. Hollingsworth had repeatedly told Chang and Eng that, in his opinion, the death of one did not necessarily compromise the life of the other; that he could separate them by cutting close to the body of the dead one, without killing the living one. It would appear possible, in view of this, that the death of Eng was not simply the result of fright.

Shortly after the death of the Siamese Twins, Dr. William Pancoast requested the Mayor of Philadelphia to telegraph to the Mayor of Greensboro, North Carolina, in regard to the possibility of a post-mortem examination being obtained. To this the Mayor of Greensboro substantially replied that he had neither knowledge nor power in the matter. When Dr. Hollingsworth, *en route* North, arrived at Greensboro, the telegram of Dr. Pancoast was handed to him. On the evening of his arrival at Philadelphia, (Friday), he saw Dr. Pancoast and Prof. Goss, and a letter was written to the wives of the twins, proposing that Dr. Pancoast should come on to embalm and examine the bodies.

On Sunday Dr. Hollingsworth saw Prof. John Neill, and, on consultation, it was concluded that the matter was of public importance, and should not be confined to any single private individual. As the promptest method, it was deemed best to call a meeting of such physicians as were interested in the matter and could be hastily got together.

The meeting took place on the evening of Monday, January 26, 1874, at the house of Dr. Neill; but, although a number had been asked, only Prof. Leidy and Dr. Rushenberger, besides Drs. Hollingsworth and Neill, were present at the conference. As the result of their deliberations, it was determined that two gentlemen should be at once dispatched to the homes of the twins, in order to examine and embalm the bodies as speedily as possible; and it was agreed that Drs. William H. Pancoast and Harrison Allen should be requested to go.

It will be seen at once that the College of Physicians was in no wise responsible for the acts of the Commission, although its name was freely used by the prominent Fellows engaged in the transaction. Indeed, these gentlemen, recognizing this, were prepared to meet the expenses of the trip had the College failed to indorse their action.

Owing to various obstacles and embarrassments, the Commission did not leave the city until Thursday night, January 29.

At the request of Dr. Pancoast, Dr. Andrews went with the party as a companion and aid.

The Commission arrived at Mt. Airy on the evening of Saturday, January 31, and proceeded to the residence of Eng the following morning, in company with a photographer and Dr. William Hollingsworth, who is the family physician in the absence of Dr. Joseph Hollingsworth. The widows of the twins received the Commission hospitably, and a conference was at once entered into, at which the "Mistresses" Bunker, the Commission, Dr. Hollingsworth and the widows' legal adviser were present. It was then agreed that, under consideration of embalming the bodies of the twins, permission would be granted to exhume and examine the structures distinguishing them, provided that no incisions should be made which would impair the external surface of the band. Subsequently it was agreed that limited incisions would be allowed on the posterior surface of the band. An agreement in writing was then drawn up, expressing the above restrictions, but extending authority to the Commission to remove the bodies to Philadelphia, provided that they be kept there in a fire-proof building, and held subject to the commands of the families when informed of the completion of the embalming process.

The object of the visit of the Commission, having been noised about the country, had attracted a crowd of curious people, who were willing enough to give the necessary aid in exhuming the bodies. The circumstances attending this were briefly as follows. The bodies were buried in the cellar of Eng's house, in a shallow grave, which had been covered with a tumulus of powdered charcoal. This being removed revealed several planks covering an outer wooden box, which, in turn, enclosed a tin encasement to the coffin. After unsoldering the tin box, the coffin was carried to the second floor of the house, to a large chamber. The lid was unscrewed, and the object of the search of the Commission was exposed to view. It was certainly an anxious moment. Fifteen days had elapsed since death, and no preservative had been employed. It was an agreeable surprise, therefore, that no odor of decomposition escaped into the room, and that the features gave no evinence of impending decay. On the contrary, the face of Eng was that of one sleeping; and the only unfavorable appearance in Chang was a slight lividity of the lips and a purplish discoloration about the ears. The widows at this point entered the room, and, amid the respectful silence of all present, took a last look at the remains.

The room was then cleared of all not connected with the work of the Commission; the bodies were disrobed, and preparations at once begun to secure photographs. The bodies were held in an erect position, and negatives of the entire figures, and views of the band at short foci, were secured. The day being cloudy, much time was necessarily expended in obtaining these pictures, time sufficient for a number of observations upon the external appearance of the bodies to be recorded. The notes are given just as they were taken at the time:

*Examination made Sunday, February* 1, 1874, *fifteen days and eight hours after the death of Chang.*

The bodies were found in the coffin in a good state of preservation; there was a slight cadaveric odor about Chang, with marked passive congestion of the back of the arms and neck on both sides, and in a less degree of the posterior aspect of the fore-arms, buttocks, thighs, and legs; there was none on the feet, where, however, there was marked fulness of the superficial veins; this was better marked on the left side. There was a greenish discoloration on the anterior abdominal wall.

*Face.*—Lips moist and discolored; peculiar reddish congestion sparsely distributed over malar prominence and beneath ear. The thoracic discoloration was much deeper on the side next to Eng.

The left nipple was visible in front well towards the middle line, the right one just showing. The fingers of the right hand—the paralyzed side—were forcibly flexed, although rigor mortis was absent.

In Eng there was passive congestion of back, most marked on buttocks and infra-spinous spaces—none on the front of the body; slight greenish discoloration of anterior abdominal wall.

In both subjects the hair of the head was gray.

On *the pubis* of each subject the hair of the *left side* was *gray*, that of the *right side, black.*

The process of embalming was now begun. Incisions were made to the outer side of the median line of the abdomen in each individual, extending from the inferior margin of the thorax to a point midway between the symphysis pubis and the anterior puperior spinous process of ilium. The aorta was reached after the usual method, but was found to be in atheromatous condition, compelling the selection of the left primitive iliac for the insertion of the pipe. A solution of chloride of zinc was then injected.

After the embalmment had been completed, the incision was continued upward and inward towards the band. Examination of the band through this incision convinced the Commission of the complex nature of the band, and suggested the suspension of a complete study of the parts until removal of the bodies to Philadelphia. The fact that the photograph had been far from satisfactory strengthened the Commission in its decision to stop the investigation at this stage. The incisions were, therefore, sewn up, the clothing readjusted, and the bodies placed in the coffin and conveyed to Mt. Airy. Here the tin box which was used for the temporary burial was again brought into use, and the lid carefully resoldered. Without delay the Commission started on its return, expressing the bodies at Salem.

The Commission arrived in Philadelphia, February 5, having been absent one week.

Upon the arrival of the bodies at the College of Physicians, they were placed in the care of the committee upon the Mutter Museum and of the Hall Committee, and were closely locked and guarded until a special meeting of the College was called, upon Monday evening, February 8, when, after considerable discussion, it was agreed that the College should accept the action of its Fellows and pay the expenses of the trip. Further, a vote of thanks was given to the gentlemen who went to North Carolina, and to Dr. Hollingsworth, and the Mutter Committee was authorized to appropriate three hundred and fifty dollars for the preparation of casts and photographs, which should remain the property of the Museum. Finally, the College appointed the Mutter Museum Committee and the original Commission (Drs. Pancoast and Allen) as a joint committee for carrying out the examination of the Siamese Twins; it being understood that a report and a demonstration of the specimens were to be made to a subsequent meeting of the College; also, that the dissections and the report were to be the work of the original Commission.

On Tuesday, the 10th instant, they were exposed for study: they were at that time found in a satisfactory condition, except the right lower extremity of Chang, which required immediate care to prevent further destructive changes taking place.

